# Role of vascular endothelial growth factor polymorphisms (-*2578C > A*, -*460 T > C*, -*1154G > A*, *+405G > C* and +*936C > T*) in endometriosis: a case–control study with Brazilians

**DOI:** 10.1186/1472-6874-14-117

**Published:** 2014-09-26

**Authors:** Jamila Alessandra Perini, Jessica Vilarinho Cardoso, Plínio Tostes Berardo, Rosane Vianna-Jorge, Luiz Eurico Nasciutti, Marta Bellodi-Privato, Daniel Escorsim Machado, Mauricio Simões Abrão

**Affiliations:** Laboratório de Pesquisa de Ciências Farmacêuticas, Unidade de Farmácia, Centro Universitário Estadual da Zona Oeste, Av. Manoel Caldeira de Alvarenga, 1203, Campo Grande, Rio de Janeiro, RJ 23070-200 Brasil; Programa de Pós-Graduação em Saúde Pública e Meio Ambiente, Escola Nacional de Saúde Pública, Fundação Osvaldo Cruz, Rio de Janeiro, RJ Brasil; Serviço de Ginecologia, Hospital Federal dos Servidores do Estado, Rio de Janeiro, RJ Brasil; Instituto de Ciências Biomédicas, Universidade Federal do Rio de Janeiro, Rio de Janeiro, RJ Brasil; Programa de Farmacologia, Coordenação de Pesquisa, Instituto Nacional do Câncer, Rio de Janeiro, Brasil; Departamento de Obstetrícia e Ginecologia da Faculdade de Medicina da Universidade de São Paulo, São Paulo, SP Brasil

**Keywords:** Endometriosis, Vascular endothelial growth factor, Polymorphisms, Brazilian population

## Abstract

**Background:**

Endometriosis is regarded as a complex and heterogeneous disease in which genetic and environmental factors contribute to the phenotype. The Vascular Endothelial Growth Factor (VEGF) plays important roles in the pathogenesis of endometriosis. The present study was aimed at investigating the contribution of *VEGF* polymorphisms as risk factors for the development of endometriosis. This is the first study to evaluate the combined influence of the five most common *VEGF* polymorphisms.

**Methods:**

This study was conducted at two hospitals from the Brazilian public health system, and comprised 294 women submitted to laparoscopic or laparotomy surgery: 182 patients had a histologically confirmed diagnosis of endometriosis (cases), whereas 112 had no evidence of the disease (controls). The *VEGF* polymorphisms were determined by TaqMan real-time polymerase chain reaction. The odds ratio (OR) with their 95% confidence intervals (CI) were calculated using an unconditional logistic regression model.

**Results:**

Endometriosis patients and controls did not differ regarding age distribution, whereas the body mass index was significantly lower in endometriosis patients, when compared with controls (23.1 ± 3.9 versus 27.3 ± 5.9, P < 0.001). The evaluation of gynecological symptoms, including dysmenorrhea, non-cyclic chronic pelvic pain, dyspareunia and infertility, indicates significantly higher prevalences among endometriosis cases. The variant allele *-1154A* was significantly associated with endometriosis, either considering all cases (OR: 1.90, 95% CI: 1.23–2.97), deep infiltrating endometriosis (DIE) (OR: 1.83, 95% CI: 1.16-2.90) or moderate and severe endometriosis (stages III-IV) (OR: 1.97, 95% CI: 1.21-3.19). No significant differences were found in allele or genotype distributions of the *–2578C > A*, -*460 T > C*, *+405G > C* and *+936C > T* polymorphisms between endometriosis cases and controls. A total of six haplotypes were inferred derived from four polymorphisms (-*2578C > A*, -*460 T > C*, -*1154G > A* and *+405G > C*). There was a protective association between *CCGG* haplotype and endometriosis, either considering all cases (OR: 0.36, 95% CI: 0.15–0.86), DIE (OR: 0.37 95% CI: 0.15 – 0.90) or stages III-IV (OR: 0.35 95% CI: 0.13 – 0.95).

**Conclusions:**

The present results indicate a positive association between *VEGF -1154G > A* and the risk of developing endometriosis, whereas the *CCGG* haplotype may be protective against the development of disease.

## Background

Endometriosis is a benign estrogen-dependent disease, characterized by the presence and growth of endometrial tissue outside the uterus, and represents one of the most common benign gynecological disorders nowadays [1]. This disease is associated with infertility, severe and incapacitating painful symptoms, including chronic pelvic pain, dysmenorrhea and dyspareunia [2, 3]. It has been estimated that endometriosis affects 10% of women of reproductive age, but the real prevalence may even be higher because it is often not diagnosed due to its heterogeneous clinical manifestation [4]. Endometriosis frequently produces serious effects on professional, social and marital life [5].

Despite many investigations about endometriosis, the pathogenesis of the disease remains unclear, although the predominant theory is that it is due to retrograde menstruation [6]. In addition, endometriotic lesions require an adequate blood supply to survive in their ectopic sites, and angiogenesis represents a crucial step during this process [7]. The development of new blood vessels is a complex dynamic process, which is regulated by a signal sequence of different angiogenic factors. The Vascular Endothelial Growth Factor (VEGF) is one of the most potent angiogenic factors and several authors postulated that it would be involved in the progress of the ectopic lesions in endometriosis [8, 9]. Accordingly, our group demonstrated that VEGF-induced angiogenesis is a critical aspect in the pathophysiology of this disease [10–12].

VEGF is encoded by the *VEGF* gene [13], which is polymorphic, with several single nucleotide polymorphisms (SNPs) in regulatory regions [14]. Recently, there is growing interest in investigating if *VEGF* SNPs may affect the inheritable susceptibility to endometriosis [15–18]. The results are conflicting, possibly due to the diversity of populations studied and because endometriosis is a heterogeneous disease [15]. In addition, no investigation regarding the susceptibility to endometriosis considered the combined effect of the five most studied *VEGF* SNPs (-*2578C > A*, -*460 T > C*, -*1154G > A*, *+405G > C* and +*936C > T*) in their possible haplotypes.

In the present work, we aimed to describe the frequency of alleles, genotypes and haplotypes of five *VEGF* SNPs among Brazilian women, and to evaluate their impact on endometriosis susceptibility.

## Methods

### Study population

The case–control study was approved by the Human Research Ethics Committee of the *Hospital das Clínicas – Faculdade de Medicina – Universidade de São Paulo* and of the *Hospital Federal dos Sevidores do Estado* (Protocols number 910/11 and 414/11, respectively). All participating patients (n = 294) provided written informed consent and answered a questionnaire about their demographics and preoperative painful symptoms. Data were obtained by in-person interviews at two hospitals from the Brazilian public health system, carried out from 2011 through 2013.

Patients assigned for laparoscopy or laparotomy for gynecological procedures were considered eligible. Individuals with any history or diagnosis of cancer or adenomyosis were not included, since both are angiogenesis-related pathologies [19, 20]. One hundred eighty-two patients undergoing laparoscopy (n = 174) or laparotomy (n = 8) for the diagnosis and treatment of endometriosis were enrolled as cases. The diagnosis of endometriosis, after their operative findings, was confirmed histologically, according to the presence of endometrial glands and/or stroma in the lesions. According to the revised American Fertility Society classification, 71 (39.0%) patients had minimal or mild endometriosis (stages I–II), 110 (60.4%) had moderate or severe endometriosis (stages III–IV) and 1 (0.6%) had these information missing. According to Nisolle and Donnez [21] three types of disease must be considered: superficial endometriosis (SUP), ovarian endometrioma (OMA) and DIE. The distribution of endometriotic patients according to their worst endometriotic lesion was as follows: SUP (14 patients; 7.7%), OMA (17 patients; 9.3%) and DIE (151 patients; 83.0%).

Controls (n = 112) were patients without visible endometriosis at surgery and who reported no previous diagnosis of endometriosis. In the control group, surgical laparoscopy (n = 106) or laparotomy (n = 6) was proposed in order to perform tubal ligation (n = 51) or treatment of benign diseases, such as ovarian cysts (n = 22), myoma (n = 10), hydrosalpinx (n = 8) or other reasons (n = 21).

The body mass index (BMI) was calculated as the weight (kg) divided by the square of height (m^2^). According to WHO’s expert committee [22], the weight status is classified into five groups: underweight (BMI < 18.5), normal weight (18.5 ≤ BMI ≤ 24.9), overweight (25 ≤ BMI ≤ 29.9), obesity (30 ≤ BMI < 40) and morbid obesity (BMI ≥ 40).

The present study focused specifically on objective symptoms, such as dysmenorrhea, chronic pelvic pain, deep dyspareunia and infertility. As suggested in our previous report [3], only severe and incapacitating symptoms of pain were included for statistical analysis purposes. Infertility (primary or secondary) was defined by the couple not being able to conceive after one year of regular, contraceptive-free intercourse.

### *VEGF*genotyping

Peripheral blood samples (3 mL) were collected in EDTA tubes, and DNA was extracted by using a commercial kit (Genomic DNA Extraction, Real Biotech Corporation) according to the manufacturer’s instructions. A validated TaqMan assay (VIC- and FAM-labeled) for detection of each *VEGF -2578C > A* (rs699947), -*460 T > C* (rs833061), -*1154G > A* (rs1570360), +*405G > C* (rs2010963), +*936C > T* (rs3025039) SNPs was purchased from Applied Biosystems. Table 1 summarizes the sets of probes and primers used for each analysis. PCR amplification for all SNPs was performed in 8 μL reactions with 30 ng of template DNA, 1× TaqMan Universal Master Mix (Applied Biosystems, Foster City, CA, USA), 1× each primer and probe assay, and H_2_O *q.s.* Thermal cycling was initiated with a first denaturation step of 10 min at 95°C, followed by 40 cycles of denaturation at 92°C for 15 s and annealing at 60°C for 1 min. The allele-detection process was performed on a 7500 Real-Time System (Applied Biosystems, Foster City, CA, USA) to determine the allelic discrimination.

**Table 1:** **Characterization of** ***VEGF*** **polymorphisms, probes and primers sequences for genotyping by TaqMan real time PCR**

Identified SNP	TaqMan assays	Region	Probe [SNP]	Primer
rs699947	C_8311602_10	PR	GCCAGCTGTAGGCCAGACCCTGGCA**[A/C]**GATCTGGGTGGATAATCAGACTGAC	5′-GGATGGGGCTGACT AGGTAAGC-3′
				5′-AGCCCCCTTTTCCT CCAAC-3′
rs833061	C_1647381_10	PR	GAGTGTGTGCGTGTGGGGTTGAGGG**[C/T]**GTTGGAGCGGGGAGAAGGCCAGGGG	5′-TGTGCGTGTGGGGTTGAGAG-3′
				5′-TACGTGCGGACAGGGCCTGA-3′
rs1570360	C_1647379_10	PR	AGCCCGGGCCCGAGCCGCGTGTGGA**[A/G]**GGGCTGAGGCTCGCCTGTCCCCGCC	5′-TCCTGCTCCCTCCT CGCCAATG-3′
				5′-GGCGGGGACAGGC GAGCATC-3′
rs2010963	C_8311614_10	5′-UTR	CGCGCGGGCGTGCGAGCAGCGAAAG**[C/G]**GACAGGGGCAAAGTGAGTGACCTGC	5′-TTGCTTGCCATTCCCCACTTGA-3′
				5′-CCGAAGCGAGAACAGCCCAGAA-3′
rs3025039	C_16198794_10	3′-UTR	GCATTCCCGGGCGGGTGACCCAGCA**[C/T]**GGTCCCTCTTGGAATTGGATTCGCC	5′-AAGGAAGAGGAGAC TCTGCGC-3′
				5′-TATGTGGGTGGGT GTGTCTACAG-3′

PR is Promoter Region, 5′-UTR is 5′-Untranslated Region, 3′-UTR is 3′-Untranslated Region.

### Statistical analysis

Comparisons of age and BMI in the study groups were performed using the Stundent’s *t* test, and data were presented as mean ± standard deviation (SD). Otherwise, the nominal data, such as spontaneous abortion, parity, infertility and preoperative painful symptoms, as well as the categories of BMI, were expressed as percentages and evaluated by Chi-Square Test or Fisher’s exact test, where applicable.

Deviations from Hardy–Weinberg equilibrium (HWE) were assessed by the goodness-of-fit χ2 test. *VEGF* allele frequency and genotype distribution were derived by gene counting. Allele and genotype frequencies between the groups were compared using the χ2 test or, when appropriate, the Fisher’s exact test. The haplotype patterns and linkage disequilibrium coefficients (D’ is degree of imbalance in module and R^2^ is degree of correlation) were inferred using Haploview [23], based on the algorithm of expectation and maximization [24]. The risk associations for endometriosis were estimated by the odds ratio (OR) with 95% confidence interval (95% CI). Confounding factors that could potentially influence the risk for endometriosis (P = 0.20) were taken into account in unconditional logistic regression models. All statistical analyses were conducted using Statistical Package for Social Sciences (SPSS Inc., Chicago, IL, USA) for Windows, version 15.0 and a *P* value less than 0.05 was considered statistically significant.

## Results

No significant difference was observed in the mean age between the endometriosis patients (35.8 ± 8.6) and the control group (34.5 ± 6.4). Conversely, BMI, parity, number of spontaneous abortion, infertility and all preoperative endometriosis symptoms were significantly different between the two groups (Table 2). There was a predominance of low or normal BMI values (≤24.9) among endometriosis patients (75.1%), whereas controls had a predominance of overweight or obesity (58.4%), with 3.4% of patients showing morbid obesity (BMI ≥ 40). The distribution of endometriotic patients according to the worst endometriotic lesion was as follows: DIE (151 patients; 83.0%) and not DIE (31 patients; 17.0%).

**Table 2:** **Demographics and clinical characteristics of the endometriosis patients and controls**

Variable	Controls	Endometriosis	***P***value ^b^
	No (%)	No (%)	
BMI			
<18.5	3 (3.4)	13 (7.7)	<0.001
18.5 ≤ BMI ≤ 24.9	31 (34.8)	113 (67.3)	
25 ≤ BMI ≤ 29.9	25 (28.1)	31 (18.5)	
30 ≤ BMI < 40	27 (30.3)	11 (6.5)	
≤ 40	3 (3.4)	0 (0)	
Parity			
0	21 (22.1)	116 (66.3)	<0.001
1	14 (14.7)	35 (20.0)	
2	26 (27.4)	18 (10.3)	
≤ 3	34 (35.8)	6 (3.4)	
Spontaneous abortion	21 (22.8)	22 (12.6%)	0.032
Infertility			
No	88 (92.6)	93 (53.1)	<0.001
Primary	6 (6.3)	60 (34.3)	
Secondary	1 (1.1)	22 (12.6)	
Symptom^a^			
Dysmenorrhoea	21 (22.3)	91 (51.7)	<0.001
Non-cyclic chronic pelvic pain	36 (38.3)	91 (51.7)	0.036
Deep dyspareunia	12 (12.9)	100 (57.5)	<0.001

BMI is Body mass index. ^a^A patient can have more than one concomitant symptom; ^b^Chi-Square Test or Fisher’s exact test.

**Figure 1:**
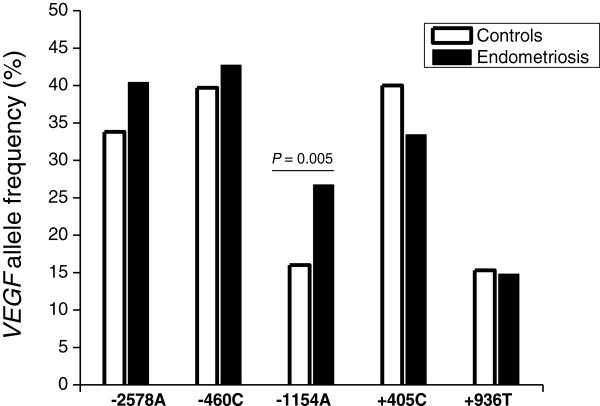
**Allelic frequencies of** ***VEGF*** **polymorphism in cases and controls.** P value from Chi-square test (Pearson p-value).

The *VEGF -2578C > A*, -*460 T > C*, -*1154G > A*, *+405G > C*, *+936C > T* SNPs were in HWE in the overall study population and in each group (cases and controls). Figure 1 and Table 3 show, respectively, the minor allelic and genotypic frequencies of the *VEGF* SNPs. Significant differences in the allele and genotype frequencies were observed between the two groups with respect to the *-1154G > A* (P = 0.005 and P = 0.01, respectively). By contrast, no significant differences were detected in allele or genotype distribution of the *-2578C > A*, -*460 T > C +405G > C*, *+936C > T* SNPs between endometriosis patients and controls. The analysis of risk associations for the *-1154G > A* in developing either endometriosis or DIE (Table 4) suggests an approximate 2-fold increased risk for individuals with any variant genotype (GA + AA), or an approximate 6-fold increased risk for individuals with the homozygous variant genotype AA. Although no statistically significant risk association was detected for individuals with the heterozygous variant genotype (GA), a codominance model was inferred for the *-1154G > A* polymorphism (P_trend_ = 0.008).

**Table 3:** **Genotypic distribution of** ***VEGF*** **SNPs in endometriosis patients and controls**

SNP	Population	N*	Genotypic distribution N (%)			P χ ^2^
**-2578C > A**			**CC**	**CA**	**AA**	
	Controls	111	50 (45.0)	47 (42.3)	14 (12.7)	0.19
	Cases	178	61 (34.3)	90 (50.6)	27 (15.1)	
**-460 T > C**			**TT**	**TC**	**CC**	
	Controls	107	39 (36.4)	51 (47.7)	17 (15.9)	0.50
	Cases	179	54 (30.2)	97 (54.2)	28 (15.6)	
**-1154G > A**			**GG**	**GA**	**AA**	
	Controls	106	74 (69.8)	30 (28.3)	2 (1.9)	0.01
	Cases	161	90 (55.9)	56 (34.8)	15 (9.3)	
**+405G > C**			**GG**	**GC**	**CC**	
	Controls	110	38 (34.6)	56 (50.9)	16 (14.5)	0.16
	Cases	181	83 (45.9)	75 (41.4)	23 (12.7)	
**+936C > T**			**CC**	**CT**	**TT**	
	Controls	95	67 (70.5)	27 (28.4)	1 (1.1)	0.63
	Cases	165	120 (72.8)	41 (24.8)	4 (2.4)	

N* is the number of examined samples of cases and controls for each SNP. Differences in sample sizes are due to available data from PCR amplification for each SNP. P χ2 is P from Chi-square test (Pearson p-value) or Fisher’s exact test.

**Table 4:** **Association analyses of the -1154G > A** ***VEGF*** **polymorphism in endometriosis patients compared with women without disease**

-1154G > A	Controls	Cases	OR (95% IC) ^b^	DIE Cases	OR (95% IC) ^c^	Stages III-IV	OR (95% IC) ^d^
	(n = 106)	(n = 161)		(n = 131)		(n = 97)	
	N (%)	N (%)		N (%)		N (%)	
**Genotypes**							
**GG**	74 (69.8)	90 (55.9)	1^a^	75 (57.3)	1^a^	56 (57.7)	1^a^
**GA**	30 (28.3)	56 (34.8)	1.54 (0.90 - 2.63)	44 (33.6)	1.45 (0.82 - 2.54)	29 (29.9)	1.28 (0.69 - 2.37)
**AA**	2 (1.9)	15 (9.3)	6.17 (1.37 - 27.8)	12 (9.1)	5.92 (1.28 - 27.4)	12 (12.4)	7.93 (1.70 - 36.9)
**Non-GG (GA + AA)**	32 (30.2)	71 (44.1)	1.82 (1.09 - 3.06)	56 (42.7)	1.73 (1.01 - 2.96)	41 (42.3)	1.69 (0.95 - 3.02)
**Allele**							
**G**	178 (84.0)	236 (73.3)	1^a^	194 (74.1)	1^a^	141 (72.7)	1^a^
**A**	34 (16.0)	86 (26.7)	1.90 (1.23 - 2.97)	68 (25.9)	1.83 (1.16 - 2.90)	53 (27.3)	1.97 (1.21 - 3.19)

OR is odds ratio, CI is confidence interval. ^a^Reference Group; ^b^Controls vs. Cases (All patients with endometriosis); ^c^Controls vs. Deeply infiltrating endometriosis patients (DIE); ^d^Controls vs. Moderate or severe endometriosis patients (stages III or IV). Due to insufficient DNA samples, some of the patients were not genotyped for -1154G > A SNP.

**Figure 2:**
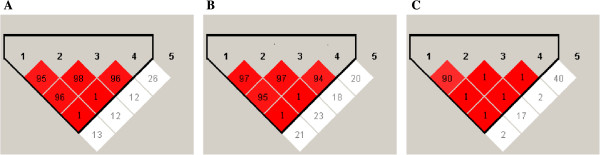
**Haplotype association analysis for the five** ***VEGF*** **polymorphisms in a Brazilian women.** Number in boxes indicates decimal places of D’. **A** Haplotype association analysis in all patients Brazilian women; **B** cases with endometriosis and **C** controls were estimated utilising Haploview program based on the Expectation-Maximization algorithm. There was a strong linkage disequilibrium patterns across the -2578C>A (*lane 1*), -460T>C (*lane 2*), -1154G>A (*lane 3*) and +405G>C (*lane 4*) *VEGF* SNPs of the three studies groups, while +936C>T (*lane 5*) was not linked to the other polymorphisms.

Haplotypes of the *VEGF* gene were determined for all patients and also for endometriosis cases and controls separately. The results revealed that SNPs -*2578C > A*, -*460 T > C*, -*1154G > A* and *+405G > C* were in strong linkage disequilibrium, forming a single haploblock, while +*936C > T* was not linked to the other SNPs (Figure 2). Therefore, haplotype analysis was only conducted between *VEGF -2578C > A*, -*460 T > C*, -*1154G > A* and *+405G > C* SNPs, and six haplotypes were inferred (Table 5). There was negative risk association for the development of endometriosis for the haplotypes *CCGG* and *ATGG*, when compared with the reference haplotype *CTGG*, either considering all cases, only DIE patients or stages III-IV of endometriosis. In addition, the haplotype *ATGG* showed negative risk associations for the development of endometriosis when considering all cases or DIE, but not stages III-IV, whereas the haplotype *CTGC* was protective only for the development of stages III-IV.

**Table 5:** **Haplotype distributions of** ***VEGF p*** **olymorphisms in cases and controls and their association with the risk of developing endometriosis**

-2578C > A/ -460 T > C/ -1154G > A/ +405G > C ***VEGF***haplotypes	Controls	All Cases	***P***value ^b^	OR (95% CI) ^c^	DIE Cases	***P***value ^b^	OR (95% IC) ^d^	Stages III-IV	***P***value ^b^	OR (95% IC) ^e^
	(N = 112)	(N = 182)			(N = 151)			(N = 110)		
								N (%)		
					N (%)					
	No (%)	No (%)								
CTGG	42 (18.7)	85 (23.4)		1^a^	69 (22.9)		1^a^	55 (25.0)		1^a^
CTGC	90 (40.1)	121 (33.2)	0.10	0.66 (0.42 – 1.05)	104 (34.4)	0.18	0.70 (0.44 – 1.13)	69 (31.3)	0.05	0.58 (0.35 – 0.97)
ACAG	36 (16.0)	96 (26.4)	0.38	1.32 (0.77 – 2.24)	78 (25.8)	0.40	1.32 (0.76 – 2.29)	58 (26.4)	0.58	1.23 (0.69 – 2.19)
ACGG	37 (16.5)	51 (14.0)	0.23	0.68 (0.39 – 1.19)	42 (13.9)	0.27	0.69 (0.38 – 1.24)	31 (14.1)	0.21	0.64 (0.34 – 1.19)
CCGG	15 (6.7)	11 (3.0)	0.03	0.36 (0.15 – 0.86)	9 (3.0)	0.05	0.37 (0.15 – 0.90)	7 (3.2)	0.05	0.35 (0.13 – 0.95)
ATGG	4 (2.0)	0 (0.0)	0.03	-	0 (0.0)	0.05	-	0 (0.0)	0.09	-

OR is odds ratio, CI is confidence interval. ^a^Reference Group; ^b^Chi-Square Test or Fisher’s exact test; ^c^Controls vs. Cases (All patients with endometriosis); ^d^Controls vs. Deeply infiltrating endometriosis patients (DIE); ^e^Controls vs. Moderate or severe endometriosis patients (stages III or IV).

## Discussion

The pathogenesis and the molecular mechanisms that underlie the development of endometriosis have troubled investigators through many years, remaining an enigma. Endometriosis is regarded as a complex trait in which genetic and environmental factors contribute to the disease heterogeneous phenotype. Regarding the epidemiological evaluation of the study population, we observed that women with endometriosis have lower BMI and are less frequently obese than control subjects. Our results corroborate previous findings [25–29], although the reason for inverse correlation between BMI and endometriosis risk is still unclear. It can be hypothesized that genetic factors contributing to endometriosis may also be linked to BMI [30, 31]. Although epidemiological data can be used to better understand the endometriosis, further studies should investigate the genetics, environmental and physiopathological basis of the decreased BMI in women with endometriosis.

Because angiogenesis represents a critical step in the establishment and pathogenesis of endometriosis, this process has been viewed as a potential new target to better define the mechanisms that cause the disease. A large number of studies have observed that VEGF was significantly higher in women with endometriosis, which supported a key role for VEGF in the pathological angiogenesis in endometriosis [9–11]. Polymorphisms in *VEGF* may alter protein concentrations, influence the process of angiogenesis and relate to inter-individual variation in the risk of endometriosis. The promoter, and the 5’- and 3’- UTR of the *VEGF* gene contain key regulatory elements, which contribute to the high variability in VEGF production among tissues [14, 32, 33].

The inheritable susceptibility to endometriosis justifies the growing interest in identifying genetic polymorphisms that could lead to an increased risk or severity of the disease, in order to provide additional support for treatment planning. The present results indicate a positive association between *VEGF -1154G > A* and the risk of developing endometriosis, which is maintained when considering only the cases of DIE or stages III-IV. Such risk association was not observed previously [34–36]. Thus, Liu et al. [34] proposed that the *-1154AA* genotype decreased endometriosis risk compared to the *-1154GG* genotype, whereas the latter reports showed no difference in the distribution of *VEGF -1154G > A* genotypes between cases and controls [35, 36]. Nevertheless, recent studies suggest that the *VEGF -1154G > A* SNP poses an increased risk of recurrent spontaneous abortion [37, 38]. Because such studies did not evaluate the occurrence of endometriosis as a possible cause of the recurrent spontaneous abortions, it cannot be excluded as a confounding factor in the association analyses. In addition, it has been reported that the frequency of the *VEGF -1154G > A* SNP in Brazilians might be different between individuals self-identified as “Blacks” or “Whites” [39]. The present study did not collect information on race or skin color. However, all individuals came from the same region of Brazil, had similar social backgrounds, and were recruited at two public hospitals, when assigned for laparoscopic procedures, regardless of the therapeutic indication. Therefore, no major racial or color differences is expected between cases and controls, which had equal access to the public health system.

With regards to the other four *VEGF* SNPs (-*2578C > A, -460 T < C*, *+405G > C, +936C < T*), our results suggest no significant effect on the susceptibility to endometriosis. It is noteworthy that our result is in agreement with Zhao and colleagues [40], which suggested no evidence for an association between endometriosis and the *VEGF* -*2578C > A, -460 T < C, +405G > C* and *+936C < T* SNPs, when considered together in a larger number (958 cases and 959 controls) of Australian women. Such findings appear to be corroborated by other studies which evaluated the different *VEGF* SNPs independently from their effect on the risk of endometriosis in different populations, and found no significant associations with -*2578C > A* [16, 40], -*460 T < C* [16, 34, 40–48], *+405G > C* [16, 35, 40, 43, 44, 49, 50] or *+936C < T* [34, 35, 40, 51]. Nevertheless, results from a meta-analysis suggest that the *VEGF -2578C > A* might be protective for the development of endometriosis [18], whereas +*936C > T* was pointed as a risk factor [16–18]. The increased risk of endometriosis for *+936C < T* was found independently on a single study, although the SNP showed no correlation with *VEGF* mRNA in endometriosis lesions or VEGF protein levels in peritoneal fluid [44]. Accordingly, Kim and colleagues [51] showed a lack of association between *+936C < T* genotypes and serum VEGF levels in endometriosis patients and controls.

The discrepancies between different studies involving the impact of *VEGF* SNPs on the susceptibility to endometriosis may be caused by different allele frequencies and heterogeneity in the study populations, besides environmental backgrounds. A strong point of our study is that all patients recruited (cases and controls) were surgically evaluated to explore for endometriosis. The histological confirmation of endometriosis was required to define cases, whereas controls had no visible ectopic endometrium sites to excluding possibly asymptomatic endometriosis. As a limitation, our controls included women with other non-endometriosis gynecological diseases, and might provide lower risk estimates if they are also associated with the polymorphisms under study.

As far as we know, the present work is the first study to focus on the possible contribution of the five most studied *VEGF* SNPs (-*2578C > A*, -*460 T > C*, -*1154G > A*, *+405G > C* and *+936C < T*) and its haplotypes on the susceptibility of endometriosis. In agreement with previous studies, -*2578C > A*, -*460 T > C*, -*1154G > A* [34] and *-2578C > A*, -*1154G > A*, *+405G > C* [35] were in linkage disequilibrium, while the *+936C < T* was visibly physically far, and had low LD with the other 4 markers in the gene [34, 35]. Only three studies reported association between *VEGF* haplotypes and susceptibility to endometriosis; however, the haplotypes with only two [41, 46] or three SNPs [34, 35] were evaluated. In the present study, we observed negative risk associations with the development of endometriosis for the haplotypes *CTGC* (only for stages III-IV), *ATGG* (for all cases combined or DIE), and *CCGG* haplotype (for all conditions). The haplotype *ACAG*, which was the only one containing the *-1154A* allele showed a non-significant positive risk association for endometriosis, in all conditions evaluated. Taken together, the results suggest that the effects of *VEGF* haplotypes in the risk of endometriosis are more significant and clinically relevant than those of each SNP evaluated separately. It is becoming increasingly important to derive data from different populations to build a database which can then be used in future investigations to a better understanding of the genetic and environmental factors affecting risk to development endometriosis.

## Conclusion

In conclusion, our findings with *VEGF* SNPs and endometriosis in Brazilian women indicate a risk association for the polymorphism *-1154G > A*, and protective effect for the haplotype *CCGG*. This is the first study to evaluate the combined influence of the five most common *VEGF* SNPs. Therefore, further studies on the functional relevance of the *VEGF* polymorphisms and exposure to environmental factors in endometriosis are required to confirm our observations.

## Abbreviations

BMI: Body mass index
CI: Confidence interval
DIE: Deeply infiltrating endometriosis
HWE: Hardy–Weinberg equilibrium
OR: Odds ratio
SD: Standard deviation
q.s: quantum sufficit
SNPs: Single-nucleotide polymorphisms
VEGF: Vascular endothelial growth factor.
